# Mitogenomes reveal multiple evolutionary units and low genetic diversity of the critically endangered pancake tortoise *Malacochersus tornieri*

**DOI:** 10.1016/j.isci.2026.115142

**Published:** 2026-02-25

**Authors:** Chuan Jiang, Nassoro Mohamed, Rudolf Mremi, Xuda Liu, Gabriel Mayengo, Yang Liu, Reginald T. Mwaya, Wenwen Zhu, Yiming Gao, Bo Li

**Affiliations:** 1 College of Wildlife and Protected Area, Northeast Forestry University, Harbin 150040, China; 2 College of African Wildlife Management, Mweka, Moshi P.O Box 3031, Kilimanjaro, Kibosho Magharibi, Tanzania; 3 School of Ecology, Sun Yat-Sen University, Shenzhen 518107, China; 4 Department of Geography, King’s College London, London WC2B 2BG, UK; 5 College of Life Science and Technology, Jinan University, Guangzhou 510632, China; 6 State Forestry and Grassland Administration Detecting Center of Wildlife, Harbin, 150040, China

**Keywords:** Zoology, genomics, evolutionary biology

## Abstract

Pancake tortoise (*Malacochersus tornieri*), a critically endangered East African endemic, is threatened by habitat destruction and illegal collection. Its fragmented range and isolated populations may have driven genetic differentiation that could inform its conservation. We infer evolutionary histories and population structure of *M. tornieri* using mitochondrial genomes (mitogenomes) of 60 free-ranging individuals from 11 localities across northern and central Tanzania*. M. tornieri* has rearranged mitogenome structure, translocation mutations of protein-coding genes, and extremely low mitogenome nucleotide diversity. Northern and central Tanzanian populations exhibit shallow divergence and contrasting demographic histories, with recent expansion in the north and contraction in the central population. This, combined with isolation-by-distance patterns found, suggests poor population connectivity. We found two divergent clades, one in central Tanzania and the other in Kenya, with the latter diverging ∼5.74 million years ago, suggesting possible cryptic speciation. Our findings provide insights into the population status of *M. tornieri* and inform conservation management.

## Highlights

• Pancake tortoises exhibit extremely low mitogenomic genetic diversity
• Northern populations expanding, central contracting; poor connectivity across Tanzania
• Deep split ∼5.74 mya between Kenyan and Tanzanian clades suggests possible cryptic speciation
• Findings guide population-specific conservation management and habitat protection

## Graphical abstract

**Figure:**
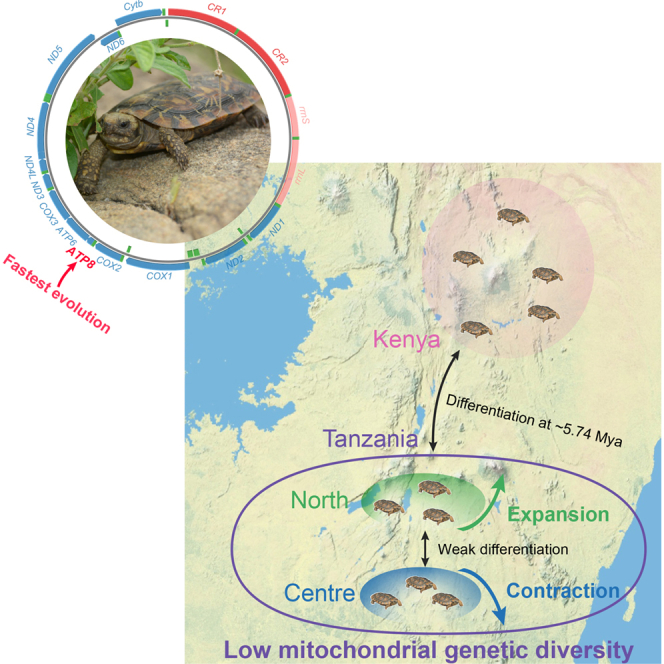

## Introduction

Human activities have triggered the sixth mass extinction, causing unprecedented decline in global biodiversity.^1^^,^^2^^,^^3^ Many species have already gone extinct, while numerous others have experienced drastic population reductions and are on the brink of extinction.^4^ Major drivers include habitat loss, climate change, spread of invasive species, pollution, and overexploitation of biological resources.^5^^,^^6^ As the foundation for adaptation and resilience, greater genetic diversity leads to greater population adaptability and viability.^7^ However, genetic diversity is being lost worldwide, representing one of the greatest challenges to biodiversity.^8^ Most endangered species with restricted habitats exhibit low genetic diversity, which reduces reproductive success and adaptive capacity, thereby elevating their extinction risk.^9^^,^^10^^,^^11^ Thus, assessing the genetic diversity of endangered species is crucial for evaluating the status of their populations. On the other hand, conservation efforts should prioritize the preservation of genetic diversity using tools such as evolutionarily significant units (ESUs) and management units (MUs). ESUs emphasize long-term evolution, while MUs emphasize short-term management.^12^^,^^13^ These units help identify and protect unique lineages and locally adapted populations, ensuring that conservation strategies target biologically meaningful groups.^14^^,^^15^

**Figure 1:**
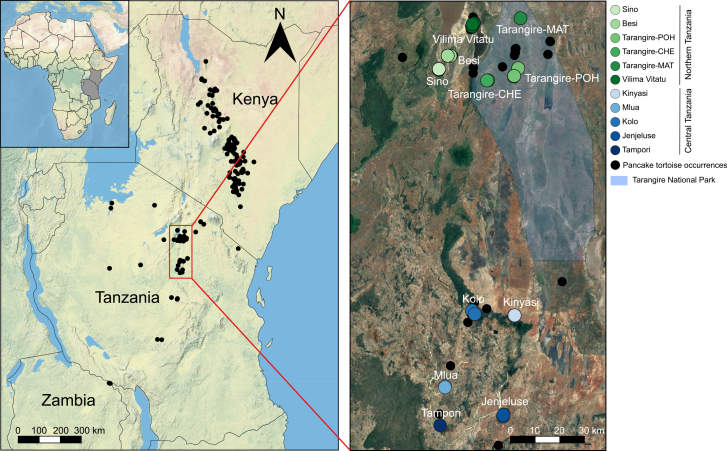
Distribution range of the *M. tornieri* and sampling sites in this study The left map shows the natural distribution of the *M. tornieri* in East Africa (Tanzania, Kenya, and Zambia), based on occurrence data obtained from various sources iNaturalist and Global Biodiversity Information Facility (GBIF), as well as relevant literature and field surveys. The right map is an enlarged satellite image of the area outlined by the red rectangle in the left map, showing 11 sampling sites for *M. tornieri* in northern and central Tanzania.

With the tortoises being among the most threatened vertebrates worldwide,^16^ understanding and preserving their genetic diversity demand urgent attention. Pancake tortoise *Malacochersus tornieri* (Siebenrock, 1903) is one of the most threatened chelonian species and is listed as a critically endangered species by IUCN.^17^^,^^18^ The species is endemic to East Africa (Figure 1) and is currently recognized as the sole extant species within its genus.^17^ Genetics studies have shown that *M. tornieri* is closely related to Asian *Indotestudo* and Eurasian *Testudo*.^19^^,^^20^^,^^21^ Pancake tortoises exhibit unparalleled morphological, behavioral, and ecological uniqueness among extant Testudinidae tortoises.^22^^,^^23^^,^^24^ Its shell shows remarkable variation in both keratinous scutes and bony elements, particularly in the number of peripheral and suprapygal bones, and the bony shell is highly fenestrated, likely reflecting adaptation to a rock-dwelling lifestyle.^25^ These specialized traits, including the tortoise’s soft, flattened shell, have also made it a target in the international pet trade.^17^^,^^18^ Illegal exploitation for the exotic pet trade has significantly reduced *M. tornieri* populations in the wild and has resulted in local extirpation of some populations.^17^^,^^18^ To control trading in this species, its status was elevated to the “Appendix I” category in the Convention on International Trade in Endangered Species of Wild Fauna and Flora (CITES) in 2019 (CITES, https://www.cites.org). With only 23% of *M. tornieri* suitable habitat protected, the species is under continued threats from habitat destruction, agriculture expansion, and climate change.^26^^,^^27^^,^^28^ Moreover, *M. tornieri* distribution is not continuous, and the subpopulations are scattered across a few rocky outcrops in Kenya and Tanzania, with one population in only one locality in northern Zambia,^17^^,^^22^^,^^23^ which may have resulted in undetected genetic differentiation (i.e., multiple ESUs).^17^^,^^29^ Despite the ecological uniqueness of *M. tornieri*, the species remains largely understudied, with limited understanding of genetic diversity and connectivity among disjunct populations hindering effective conservation efforts.^17^^,^^30^

The application of molecular techniques in genetic studies allows conservation practitioners to precisely determine the status of endangered species, providing important insights for species management.^31^ Among the molecular markers, the mitochondrial genome (mitogenome) has become an important marker in conservation genetics, taxonomy, and phylogenetics due to its conserved gene organization, relatively fast and predictable evolutionary rate, maternally linked inheritance, lack of recombination, and relative ease of amplification, although it also has some well-recognized limitations.^32^^,^^33^^,^^34^ For example, studies across multiple species demonstrate that mitogenomes offer essential data on genetic diversity, directly informing conservation strategies and supporting targeted conservation.^10^^,^^35^^,^^36^ In addition, because the mitogenomes have more copy numbers than nuclear genes, they typically offer an advantage in non-invasive sampling studies of endangered species, although modern nuclear DNA methods can also work with minimal tissue input. Therefore, research on mitogenomes contributes to the preliminary assessment of the genetic structure, diversity, and demographic history of endangered species, thus revealing population connectivity and identifying MUs.^31^

In this study, we sequenced and assembled mitogenomes of 60 *M. tornieri* individuals from 11 localities across northern and central Tanzania (Table S1) and performed population genetic analyses in combination with one published mitogenome. We first characterized the population evolutionary features of these mitogenomes and then revealed the genetic structure, genetic diversity, and recent demographic history of *M. tornieri*. We also assessed the divergence between Tanzanian and Kenyan populations based on the mitochondrial gene *COX1*. The findings of this study will help to inform conservation strategies and population management for this critically endangered species.

## Results

### Distinct mitogenomic configuration

**Figure 2:**
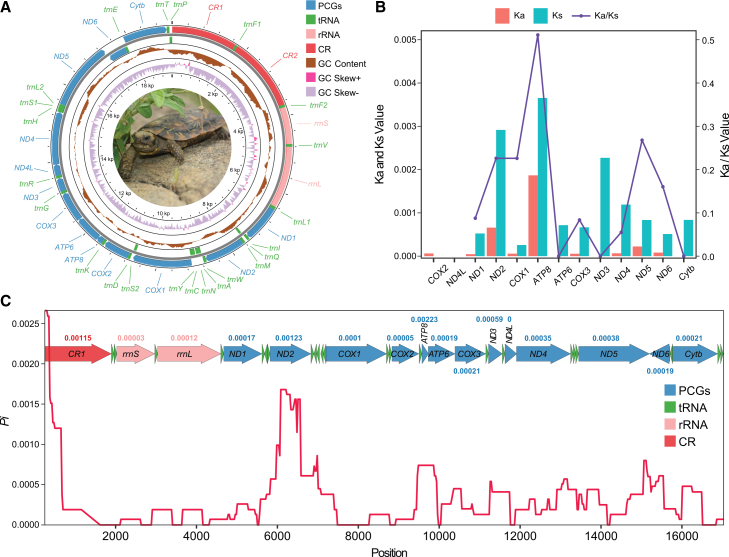
Mitogenome structure and evolutionary features of *M. tornieri* (A) The circle map illustrates the unique mitogenome structure of *M. tornieri*. Genes encoded on the H or L strand are shown on the outside or inside of the map, respectively. The GC content (500 bp window size) indicates deviations from the GC content across mitogenome. The GC skew (500 bp window size) is plotted using a red and purple sliding window, representing positive and negative values, respectively. (B) The Ka, Ks, and Ka/Ks values of the 13 PCGs suggest their evolution under purifying selection. (C) The *Pi* values of the mitogenome (excluding *CR2*) plotted using a sliding window (500 bp window size with 25 bp step size) reveal higher variability in the *CR1* and *ND2*. The arrows above the line graph represent genes, with their direction indicating gene orientation. The numbers above or below the arrows (color-matched) correspond to the *Pi* values of the respective genes, with only the longer genes’ *Pi* values listed.

Mitogenomes were assembled from quality-controlled reads. Sample MAL100 yielded identical circular mitogenomes from both HiFi long-read and PE150 short-read assemblies. The remaining 59 samples also yielded circular assemblies, with sequencing depth plots confirming structural integrity (Figure S1). All mitogenomes exhibited a conserved structure, consisting of 13 protein-coding genes (PCGs), 2 *rRNAs*, 2 *CRs*, and 23 *tRNAs* (Figure 2), consistent with previous reports for this species.^19^ This structure features an additional *CR* and tRNA-Phe (*trnF*) compared to other Testudinidae mitogenomes, which can be explained by the tandem duplication and random loss (TDRL) model.^37^^,^^38^

Mitogenome lengths ranged from 18,136 to 19,634 bp (Table S2), primarily due to variation in repeat sequences in *CRs*. This was further validated by sequencing depth plots of reads from each sample mapped to MAL100 mitogenome, where some samples exhibited no read coverage in certain *CR* regions (Figure S2). The lengths of the 13 PCGs were conserved across all samples, with no insertions or deletions detected, ranging from 297 bp (*ND4L*) to 1,803 bp (*ND5*). Start and stop codons were also conserved, with initiation codons including ATG, GTG, and CTG and termination codons including TAG, AGG, TAA, and T (Table S3). Consistent with previous findings,^19^ adenine insertions were identified at position 175 of *ND3* and at position 172 of *ND4*, conserved in all samples and likely tolerated through RNA editing or a specialized mitochondrial translation system.^19^^,^^39^ Except for *COX2* (no non-synonymous substitutions) and *ND4L* (no substitutions), all PCGs had Ka/Ks ratios <1 (Figure 2B), indicating purifying selection, possibly related to the high metabolic demands of their rapid mobility. *ATP8* exhibited the highest synonymous and nonsynonymous substitutions, consistent with previously reported evolutionary rates in birds and reptiles.^40^ Sliding window analysis of *Pi*, excluding poorly aligned *CR2*, revealed heterogeneous polymorphism, with *CR1*, *ND2*, and *ATP8* showing markedly elevated *Pi* values, *ATP8* being the most variable (Figure 2C).

### Shallow divergence between central and northern Tanzanian populations and low genetic diversity

**Figure 3:**
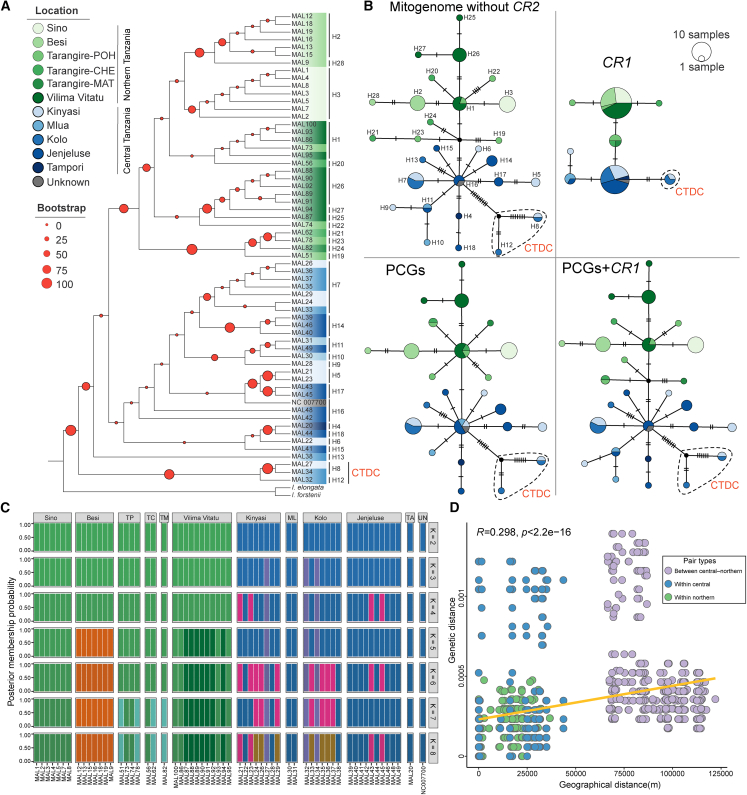
Genetic structure and Mantel test of the *M. tornieri* population (A) Individual ML phylogenetic tree constructed based on mitogenomes without *CR2*. Haplotypes are listed on the right side of the sample labels. The red-marked CTDC represents three highly divergent samples. (B) MJ haplotype networks based on different mitogenome regions. The haplotypes in the mitogenome without the *CR2* network correspond to those in the phylogenetic tree (A). The dashed circle encloses the haplotype group CTDC corresponding to the three highly divergent individuals. (C) DAPC result with K set to 2–8. TP represents Tarangire-POH, TC represents Tarangire-CHE, TM represents Tarangire-MAT, ML represents Mlua, TA represents Tampori, and UN represents unknown. (D) Mantel tests revealed a significant positive correlation between genetic and geographic distances in *M. tornieri*. Green and blue dots denote sample pairs from northern and central Tanzania, respectively, while purple dots represent interregional pairs.

Phylogenetic analyses using three methods, together with MJ haplotype networks constructed from different genes, consistently revealed a clear yet shallow divergence between northern and central Tanzanian populations without mixing (Figures 3A and 3B), despite topological conflicts in some low-support nodes among the phylogenetic trees (Figures 3A and S3). Within the northern Tanzanian and central Tanzanian populations, individuals from different localities showed slight mixing. Notably, three central Tanzanian individuals (MAL27 from Kinyasi; MAL32 and MAL34 from Kolo) carried more mutations and formed a distinct clade (central Tanzania divergent clade [CTDC]) in all results (Figures 3A, 3B, and S3). Discriminant analysis of principal components (DAPC) further corroborated these findings, indicating independent ancestral components between northern and central populations regardless of K, while some localities within each area were composed of more than one ancestral component at K > 2 (Figure 3C). The CTDC individuals consistently displayed unique ancestral components at K ≥ 3. Sequences of unknown origin clustered with northern Tanzanian individuals in all analyses, indicating a probable origin in this region.

Pairwise *F*st calculations indicated minimal genetic differentiation among most localities, with only a few pairs showing significant divergence (Table S4). Pairwise *Fst* analysis revealed significant differentiation between several populations (*p* < 0.01), with the maximum value (0.00794) occurring between Kolo and Jenjeluse. Notably, no significant differentiation was detected between northern and central Tanzanian populations (*F*st = 0). Analysis of molecular variance (AMOVA) for the 11 localities indicated that variation within localities (52.98%) was greater than variation among localities (47.02%), whereas AMOVA for northern and central Tanzanian populations indicated a more pronounced difference (57.08% within compared to 42.92% among; Table S5). These results suggest weak genetic structure among localities within the northern and central Tanzanian areas.

Mantel tests on georeferenced samples revealed a significant isolation-by-distance (IBD) pattern (R = 0.298; Figure 3D), which became stronger after excluding outlier CTDC individuals (R = 0.658; Figure S4A). IBD was also significant among northern Tanzanian individuals (R = 0.474; Figure S4B). For central Tanzanian individuals, the test was not initially significant (Figure S4C) but became so after exclusion of CTDC individuals (R = 0.121; Figure S4D).

**Figure 4:**
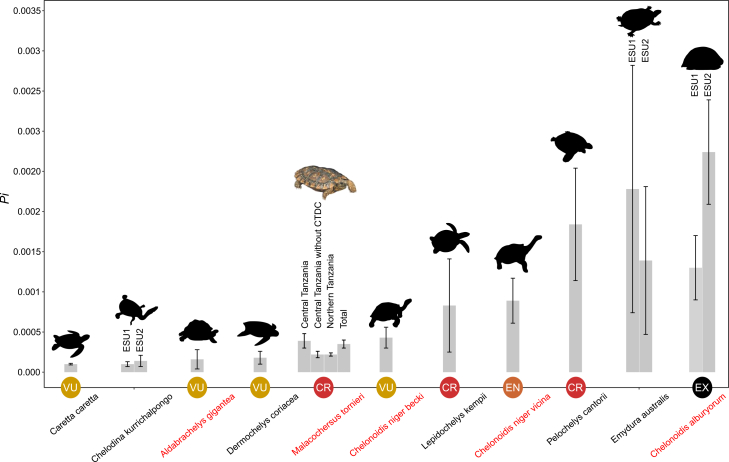
Comparison of genetic diversity among *M. tornieri* and other chelonians based on 13 mitogenome PCGs For species with genetic substructures, the *Pi* of different ESUs is shown. EX, extinct; CR, critically endangered; EN, endangered; VU, vulnerable. Species of the family Testudinidae are highlighted in red.

Genetic diversity in *M. tornieri*, calculated from the mitogenome without *CR2*, revealed 28 haplotypes among 61 individuals, with total values of *h* = 0.887, *Pi* = 0.00035, and *K* = 5.80 (Table 1). Among the 11 localities, Jenjeluse exhibited the highest *h* (0.889), while Kolo showed the highest *Pi* (0.00052) and *K* (9.05). Central Tanzania displayed higher diversity (*h* = 0.952, *Pi* = 0.00035, and *K* = 5.80) than northern Tanzania (*h* = 0.887, *Pi* = 0.00018, and *K* = 3.05). However, after excluding the outlier CTDC from central Tanzania, its diversity indices decreased (*h* = 0.907, *Pi* = 0.00017, and *K* = 2.97), becoming comparable to northern Tanzania. *Pi* calculated from 13 PCGs yielded similar results: northern Tanzania = 0.00022, central Tanzania = 0.00039, central Tanzania without CTDC = 0.00022, and total *Pi* = 0.00035 (Table S6). These values are relatively low compared with other turtles calculated using the same genes (Figure 4; Table S6).

**Table 1:** Statistics on maternal genetic diversity indices and neutrality tests of *M. tornieri* based on mitogenome without *CR2*

Population	Ns	Nh	*H*	*Pi*	*K*	*D*	*F*_*S*_
Sino	7	1	0	0	0	–	–
Besi	7	2	0.286 ± 0.039	0.00003 ± 0.00002	0.571	−1.24	0.856
Tarangire-POH	4	4	1.000 ± 0.076	0.00028 ± 0.00007	4.66667	−0.49	−0.615
Tarangire-CHE	2	2	1.000 ± 0.500	0.00024 ± 0.00012	4.00000	–	–
Tarangire-MAT	1	1	0	0	0	–	–
Vilima Vitatu	11	4	0.709 ± 0.099	0.00008 ± 0.00001	1.38182	0.043	−0.053
Kinyasi	8	5	0.857 ± 0.108	0.00042 ± 0.00017	7.25	−1.31	1.62
Mlua	2	2	1.000 ± 0.500	0.00006 ± 0.00003	1.00	–	–
Kolo	7	4	0.714 ± 0.181	0.00052 ± 0.00017	9.05	−0.432	3.16
Jenjeluse	10	6	0.889 ± 0.075	0.00015 ± 0.00003	2.53	−0.992	−1.61
Tampori	1	1	0	0	0	–	–
Northern Tanzania	32	13	0.887 ± 0.030	0.00018 ± 0.00002	3.04839	−1.54∗	−3.87∗
Central Tanzania	28	15	0.923 ± 0.035	0.00033 ± 0.00008	5.76984	−1.51∗	−3.10
Central Tanzania without CTDC	25	13	0.907 ± 0.043	0.00017 ± 0.00002	2.96667	−1.47	−5.60∗
Unknown	1	1	0	0	0	–	–
Total	61	28	0.952 ± 0.011	0.00035 ± 0.00004	5.80	−1.86∗	−10.4

Note: Ns, number of samples; Nh, number of haplotypes; *Pi*, nucleotide diversity; *h*, haplotype diversity; *K*, average of nucleotide differences; *D*, Tajima’s *D*; *Fs*, Fu’s *Fs*; ∗*p* < 0.05.

### Contrasting demographic histories between central and northern Tanzanian populations

**Figure 5:**
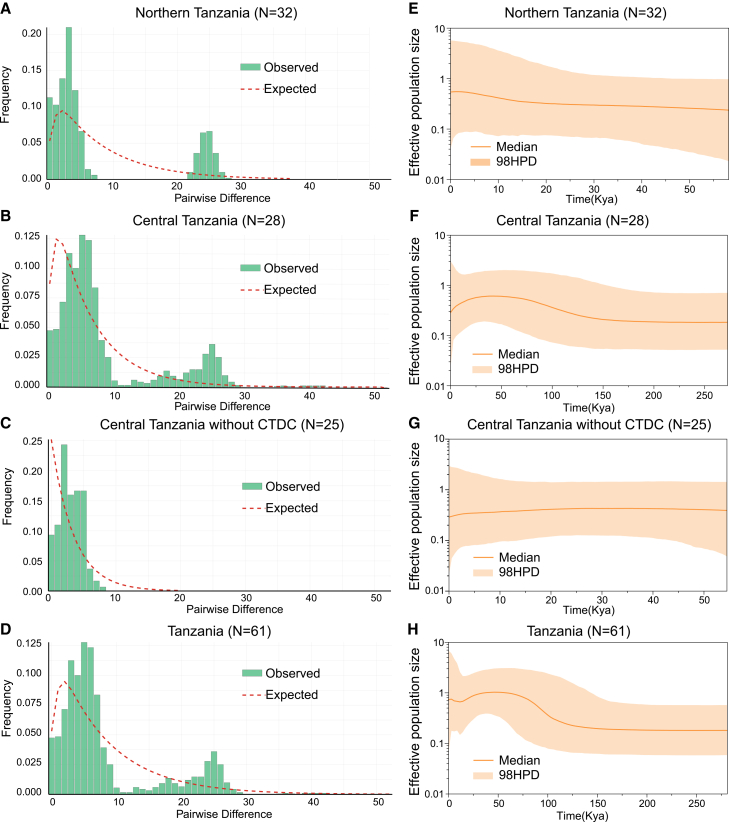
The mismatch distribution and Bayesian skyline plots reveal different demographic histories between the Tanzanian central and northern populations (A–D) Mismatch distribution based on the mitogenomes without *CR2*. (E–H) Bayesian skyline plots based on the mitogenomes without *CR2*.

Except for a few localities with insufficient sample sizes for neutrality tests, all others showed non-significant results (Table 1). In contrast, significantly negative values of Tajima’s *D*, Fu’s *Fs*, or both were observed in the northern Tanzania, central Tanzania (with and without CTDC), and the total dataset, suggesting recent demographic expansion. The mismatch distributions for northern Tanzania, central Tanzania, and total datasets were multimodal (Figures 5A, 5B, and 5D), indicating a complex demographic history in *M. tornieri*, yet all shared a dominant peak at pairwise differences below 10 (Figures 5A–5D), supporting recent expansion. Furthermore, the unimodal distribution of central Tanzania without CTDC confirms that high-difference peaks in the full central dataset were attributable to CTDC individuals (Figures 5B and 5C).

The Bayesian skyline plot (BSP) reveals a more detailed demographic history, particularly showing distinct trends between northern and central Tanzania (Figures 5E–5H). The northern population, dating back to ∼58 kya (thousand years ago), shows slow and steady growth (Figure 5E). In contrast, the demographic history of central Tanzania, which includes individuals with additional mutations in CTDC, can be traced back to ∼273 kya (Figure 5F). It initially grew slowly, began rapid expansion from ∼150 kya, and then declined ∼40 kya. Central Tanzania without CTDC traces back to a similar time as northern Tanzania, approximately 55 kya (Figures 5E and 5G), with population trends mirroring those of central Tanzania during the same period (Figures 5F and 5G). For all individuals, the demographic history dates back to ∼273 kya, remaining nearly stable initially, then experiencing rapid growth starting ∼150 kya, followed by a decline starting ∼40 kya, and a renewed increase starting ∼10 kya (Figure 5H).

### Deep divergence between Tanzanian and Kenyan populations

**Figure 6:**
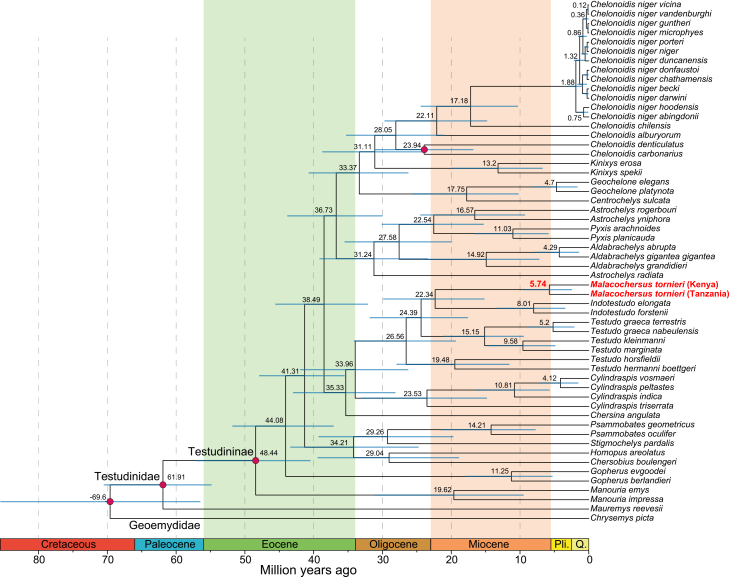
Phylogenetic tree with divergence times inferred from partial *COX1* sequences Estimated mean ages and 95% highest posterior density (HPD) intervals are shown at the nodes. Four fossil calibration points are indicated by red circles. Geological periods are indicated below the tree, where Pli. denotes the Pliocene and Q. denotes the Quaternary. The labels for *M. tornieri* from Kenya and Tanzania, along with their divergence time, are highlighted in bold red.

The *COX1* sequences of 63 *M. tornieri* (Table S7) were trimmed to 660 bp based on homologous sequences, resulting in three haplotypes: H1 for Tanzania without CTDC, H2 for CTDC, and H3 for Kenya (Figures S5A and S5B). Phylogenetic trees constructed using maximum likelihood (ML) and Bayesian inference (BI) methods revealed a deep split between the Kenyan and Tanzanian populations, which was further supported by the haplotype network (Figure 6C).

Divergence time estimation based on four fossil calibration points for 56 species/subspecies (Table S8) produced a time framework similar to previous studies,^41^ wherein *M. tornieri* from Tanzania and Kenya diverged in the Late Miocene, at 5.74 mya (95% highest posterior density [HPD] 2.41–9.45 mya) (Figure 6). Notably, this divergence predates some species-level differentiations, such as that between *Cylindraspis vosmaeri* and *C. peltastes* at 4.12 mya (95% HPD 1.51–7.17 mya) and between *Geochelone elegans* and *G. platynota* at 4.7 mya (95% HPD 1.63–8.26 mya).

## Discussion

Our findings indicate that the unique mitogenome organization reported previously for *M. tornieri*, characterized by gene rearrangements and translocation mutations of PCGs, is consistent across all sampled individuals. Despite relatively high *h* in these mitogenomes, the overall *Pi* is extremely low, which may be associated with slow mitochondrial mutation rates commonly observed in turtles, as reported in previous studies.^40^^,^^42^ However, the *Pi* remains relatively low compared to other turtles (Figure 4; Table S6), even though the *M. tornieri* may exhibit a higher mutation rate.^19^ This may result from either a historical population bottleneck, particularly during the last glacial maximum (LGM), or long-term small effective population size (*Ne*). Supporting this, the *Ne* of *M. tornieri*, particularly in central Tanzania, declined at ∼40 kya, roughly coinciding with the LGM, when cooler and drier climates contracted suitable habitats^43^ reducing *Ne*. Although patterns of high *h* and low *Pi*, significantly negative neutrality tests, low nucleotide mismatch peaks, and BSP trends indicate a recent slight expansion potentially linked to post-glacial environmental improvement,^44^ the persistently low genetic diversity (primarily *Pi*) still suggests potential threats to the long-term survival of the Tanzanian population. This raises concerns about the species’ resilience in the face of ongoing population declines in parts of its distribution range.^17^

We also found shallow divergence without mixing and distinct demographic histories between northern and central Tanzanian populations, suggesting their poor connectivity and that they warrant status as two ESUs. Furthermore, the substructure within both areas, combined with the IBD patterns observed, further indicates poor connectivity among localities and highlights the important role of geographical distance in shaping population structure. Potential barriers—such as mountains, rift valleys, rivers, roads, and human-modified landscapes—likely continue to impede dispersal and promote population differentiation^45^^,^^46^ particularly given the species’ limited vagility.^47^^,^^48^ Additionally, three individuals from central Tanzania (CTDC) exhibited significantly more mutations than others, which notably influenced overall *Pi* value and spatial genetic patterns. They may have resulted from a past population bottleneck that caused the loss of intermediate haplotypes.^49^^,^^50^^,^^51^ Alternatively, they may have been caused by ancient divergence events, with lineages persisting in these regions or originating from migration from unsampled areas, or from historic introgression after hybridization,^52^^,^^53^^,^^54^ implying the existence of highly differentiated ESUs in unsampled areas of Tanzania. Moreover, the region of central Tanzania, where Kolo and Kinyasi are located, is home to a variety of ancient rock paintings by hunter-gatherers who lived in the area for over two millennia.^55^ If the CTDC in these localities originated via migration or introgression, it could also be linked to translocation practices of these hunter-gatherers (and later, pastoralists).

Interestingly, despite the use of a single gene (*COX1*), which has a limited resolution among Tanzanian populations, it nonetheless clearly indicates a deep genetic divergence between Tanzanian and Kenyan populations during the Late Miocene (5.74 mya). The deep divergence temporally coincides with major geological and climatic events in East Africa during the Late Miocene, a period characterized by extensive tectonic activity associated with the formation of the East African rift and a shift toward increased aridity.^43^^,^^56^ Although no direct tests of climatic or geological causation were performed, the timing of these divergence events suggests that the emergence of large-scale landscape features and associated ecological transitions may have contributed to the isolation of the two ESUs. Similar temporal congruence between diversification and Late Miocene environmental change has been reported in other reptiles.^57^^,^^58^^,^^59^ Importantly, the observed divergence predates some other species-level divergences in Testudinidae, highlighting the evolutionary distinctiveness of the Kenyan and Tanzanian lineages. This raises the question of whether the Tanzanian and Kenyan populations of *M. tornieri* represent separate species.

While habitat management and control of illegal tortoise trade remain critical to enhance long-term survival of the *M. tornieri*, there is an urgent need for proactive measures to preserve the existing genetic diversity. Given the observed low genetic diversity within Tanzanian populations, the subtle but present internal population structure, and the deep divergence from Kenyan populations, we recommend integrating our findings to the region-specific conservation strategies. With the insights from our study, we recommend that, at a minimum, three MUs should be considered: the central Tanzanian population, the northern Tanzanian population, and the Kenyan population. These groups should be treated as separate MUs to preserve their evolutionary potential and uniqueness. Since the central Tanzanian population has recently faced a continuous decline in population size and possesses unique mitochondrial clades such as CTDC, this population should be given priority for conservation. In addition, given the presence of IBD patterns, conservation efforts should also consider maintaining habitat connectivity to prevent potential inbreeding depression and local extinction within localities. Furthermore, conservation actions such as protecting the breeding sites and key microhabitats, improving enforcement to protect the species, and limiting habitat loss from quarrying and wildfires are critically important. Additionally, because a large portion of the pancake tortoise population occurs outside protected areas, effective conservation will depend on effective collaboration with local communities and stakeholders to reduce illegal collection and habitat destruction.

### Limitations of the study

Despite the insights gained from this study, some limitations should be acknowledged. First, sampling coverage remains incomplete across the species’ distribution range. Based on available *M. tornieri* occurrence data, most populations in Kenya and several small, discrete populations in Tanzania have not been studied due to sampling limitations. The evolutionary uniqueness and genetic diversity of these populations remain unknown, and they may harbor cryptic ESUs or potentially be related to CTDC, with implications for conservation prioritization. Second, the molecular clock analysis is based on a single mitochondrial gene, *COX1*. While it alone may be limited by its relatively low information content and potential saturation of nucleotide substitutions, the divergence time estimates presented in our study align with those reported in a recent study using complete mitogenomes,^41^ giving credibility to our results. Furthermore, the Kenyan population was represented by only two individuals, potentially limiting its representativeness. Finally, the inherent properties of mitochondrial genes confer both strengths and limitations as a molecular marker in population genetics and phylogenetic inference.^60^^,^^61^ With the rapid advancement of high-throughput sequencing and declining costs, future studies will likely prioritize whole-genome data to achieve a more comprehensive understanding of species’ genetic backgrounds.^62^^,^^63^ Nonetheless, we provide a valuable genetic foundation *for M. tornieri*, revealing threats from low genetic diversity and complex demography in central and northern Tanzania, offering conservation insights based on the observed genetic structure and poor population connectivity within Tanzanian populations, and revealing deep divergence between Tanzanian and Kenyan populations. We recommend broader geographic sampling covering more regions of Tanzania and Kenya, as well as the use of genomic data, to fully understand the genetic structure, diversity, load, and potential cryptic species within *M. tornieri*. In addition, comparative evaluations of morphological, behavioral, and other traits, particularly between Tanzanian and Kenyan populations, are needed to determine whether these lineages represent cryptic species.

## Resource availability

### Lead contact

Requests for further information and resources should be directed to and will be fulfilled by the lead contact, Bo Li (libo_770206@nefu.edu.cn).

### Materials availability

This study did not generate new or unique reagents.

### Data and code availability

• The mitochondrial genome data of 60 *Malacochersus tornieri* generated in this study have been uploaded to NCBI under the accession numbers PX394300–PX394359.
• This paper does not report original code.
• Any additional information required to reanalyze the data reported in this paper is available from the lead contact upon request.

## Acknowledgments

This research was supported by the 10.13039/100017131National Supercomputer Center in Guangzhou and the High-performance Computing Public Platform (Shenzhen Campus) of 10.13039/501100002402Sun Yat-sen University. B.L. was supported by the National Basic Resources Investigation Program (2023FY100405). We are grateful to the College of African Wildlife Management, Mweka, for funding and supporting field data collection logistics. We also thank the Chinese Government Scholarship Council for their essential funding of this research. We acknowledge Godwin Nyerere, Joseph Naibala, and Augustino Mwageni for their assistance with field data collection.

## Author contributions

Conceptualization, C.J., N.M., R.T.M., and B.L.; data collection, N.M. and R.T.M.; investigation, C.J., N.M., and B.L.; formal analysis, C.J.; writing – original draft, C.J. and N.M.; writing – review and editing, R.M., X.L., G.M., Y.L., R.T.M., W.Z., and Y.G.; funding acquisition, B.L.; supervision, B.L.

## Declaration of interests

The authors declare no competing interests.

## Declaration of generative AI and AI-assisted technologies in the writing process

The authors reviewed and edited the content as needed and take full responsibility for the content of the publication.

## STAR★Methods

### Key resources table

**Table:** 

REAGENT or RESOURCE	SOURCE	IDENTIFIER
**Deposited data**		

The mitochondrial genome data of 60 *Malacochersus tornieri* generated in this study	This paper	NCBI under the accession numbers PX394300-PX394359
Published *M. tornieri* mitogenome	Parham et al.^19^	GenBank: DQ080042/NC_007700
*COX1* sequences of *I. elongata*	Parham et al.^19^	GenBank: DQ080043/NC_007695
*COX1* sequences of *I. forstenii*	Parham et al.^19^	GenBank: DQ080044/NC_007696
*COX1* sequences of Kenyan M. tornieri	NCBI	GenBank: MK545068
*COX1* sequences of Kenyan *M. tornieri*	NCBI	GenBank: MK545069

**Software and algorithms**		

PMAT v2.1.5	Han et al.^64^	https://github.com/aiPGAB/PMAT2
fastp v0.23.4	Chen^65^	https://github.com/OpenGene/fastp
GetOrganelle v1.7.5	Jin et al.^66^	https://github.com/Kinggerm/GetOrganelle
NOVOPlasty v4.3.5	Dierckxsens et al.^67^	https://github.com/ndierckx/NOVOPlasty
BWA-MEM v0.7.18	Jung & Han.^68^	https://github.com/kaist-ina/BWA-MEME/
SAMtools v1.21	Danecek et al.^69^	https://github.com/samtools/samtools
MITOS v2.1.9	Zea et al.^70^	https://gitlab.com/Bernt/MITOS
tRNAscan-SE v1.21	Lowe & Chan^71^	http://trna.ucsc.edu/tRNAscan-SE/
CGView	Stothard^72^	http://wishart.biology.ualberta.ca/cgview/
MAFFT v7.313	Katoh&Standley^73^	https://mafft.cbrc.jp/alignment/software/
DnaSP v6.0.7	Rozas et al.^74^	http://www.ub.edu/dnasp/
IQ-TREE v2.2.2	Minh et al.^75^	https://github.com/iqtree/iqtree2
MrBayes v3.2	Ronquist et al.^76^	http://www.mrbayes.net
MEGA v11.0.13	Tamura et al.^77^	https://www.megasoftware.net/
PartitionFinder v2.0	Lanfear et al.^78^	https://github.com/brettc/partitionfinder
POPART v1.7	Leigh&Bryant^79^	http://popart.otago.ac.nz
Arlequin v3.5	Excoffier&Lischer^80^	http://cmpg.unibe.ch/software/arlequin35/
BEAST v1.8.4	Drummond et al.^81^	https://github.com/beast-dev/beast-mcmc
LogCombiner v2.6.4	Bouckaert et al.^82^	https://github.com/beast-dev/beast-mcmc
Tracer v1.6.1	Rambaut et al.^83^	https://github.com/beast-dev/tracer
TreeAnnotator v2.6.4	Bouckaert et al.^82^	https://github.com/beast-dev/beast-mcmc
R v4.4.3	R Core Team	https://www.rproject.org/
adegenet v2.1.11	Jombart et al.^84^	https://github.com/thibautjombart/adegenet
vegan v2.7.1	Dixon^85^	https://github.com/vegandevs/vegan
geosphere v1.5.20	Karney^86^	https://github.com/rspatial/geosphere

### Experimental model and study participant details

#### Sample collection

The necessary ethical approvals and research permits were obtained from the Laboratory Animal Management and Ethics Committee of Northeast Forestry University (No. 2024054). In Tanzania, the research was permitted by the Tanzania Wildlife Research Institute (TAWIRI) and was granted approval by the Tanzania Commission for Science and Technology (COSTECH) (Permit No: 2023 -726 -NA -2023 - 750) and Tanzania National Parks (TANAPA).

We collected 60 samples from *M. tornieri* across 11 localities in northern and central Tanzania. Among these, 59 were skin tissue samples collected between 2010 and 2011, and one blood sample was collected in 2024 at Vilima Vitatu in northern Tanzania (Figure 1 and Table S1).

### Method details

#### DNA extraction and sequencing

High-throughput sequencing was performed on all individual samples. First, total genomic DNA was extracted from skin tissue and blood using an extraction kit (UElandy, Suzhou, China). High-quality DNA was then fragmented by ultrasonication and a sequencing library with an average insert size of approximately 350 bp was constructed using the MGIEasy Universal DNA Library Preparation Kit (BGI) on the DNBSEQ platform, following the manufacturer’s instructions. Paired-end sequencing (150 bp reads) was subsequently carried out on the DNBSEQ-T7 sequencer (MGI, China).

In addition, Pacific Biosciences (PacBio) sequencing was performed on the blood samples. High-molecular-weight DNA was extracted from blood using the SDS method. A SMRTbell library (average insert size = 15 kb) was prepared with the SMRTbell Template Prep Kit 1.0 (Pacific Biosciences), size-selected using Blue Pippin (Sage Science) and polymerase-bound with the Revio Polymerase Kit. After purification (SMRTbell Cleanup Beads), the library was sequenced on a Revio system (Pacific Biosciences).

#### Mitogenome assembly, annotation and characterization

For the HiFi data, we first assembled the mitogenome of sample MAL100 (Table S2) using PMAT v2.1.5.^64^ For the PE150 data, we initially performed quality filtering using fastp v0.23.4^65^ retaining only reads with a minimum length of 100 bp while keeping all other parameters at their default settings. We then assembled the mitogenomes from the cleaned reads of each sample (Table S2) using GetOrganelle v1.7.5.^66^ Additionally, NOVOPlasty v4.3.5^67^ was used for assembly to verify structural accuracy. To further validate the completeness of the assemblies, we aligned each sample's reads to its respective assembled mitogenome using BWA-MEM v0.7.18^68^ and calculated base depth using Samtools v1.21.^69^ Due to length variations observed among different samples, we also aligned all individuals’ PE150 reads to the MAL100 HiFi-assembled mitogenome using BWA-MEM v0.7.18 to assess sequencing depth and confirm the presence of structural variations.

The assembled mitogenomes were annotated using MITOS v2.1.9^70^ with tRNA predictions cross-referenced with tRNAscan-SE v1.21.^71^ The annotation of all mitochondrial genes was manually verified by comparison with the published *M. tornieri* mitogenome (DQ080042/NC_007700),^19^ whose geographic origin is unknown.

The circular mitogenome map was generated using CGView.^72^ We incorporated a published *M. tornieri* mitogenome (NC_007700) with the 60 newly assembled mitogenomes, yielding 61 in total and aligned them using MAFFT v7.313.^73^ Because *CR2* contains numerous repetitive sequences that complicate alignment, this region was excluded from downstream analyses. Nonsynonymous (Ka) and synonymous (Ks) substitution rates of the 13 protein-coding genes (PCGs) were estimated with DnaSP v6.0.7.^74^ Nucleotide diversity (*Pi*) across the mitogenome was assessed by sliding window analysis in DnaSP v6.0.7, using a window size of 500 bp and a step size of 25 bp.

#### Genetic structure, IBD test and genetic diversity in Tanzania

The mitogenomes of *Indotestudo elongata* and *I. forstenii*^19^ were downloaded from NCBI to use as outgroups for the phylogenetic analysis of the 61 *M. tornieri* sequences. After alignment using MAFFT v7.313, phylogenetic trees were constructed from the mitogenome without *CR2* using maximum likelihood (ML) in IQ-TREE v2.2.2,^75^ Bayesian inference (BI) in MrBayes v3.2,^76^ and maximum parsimony (MP) in MEGA v11.0.13.^77^ The optimal partitioning and nucleotide substitution models for ML and BI (Table S9) were selected with PartitionFinder v2.0^78^ under the corrected Akaike information criterion (AICc). Node support was assessed using posterior probabilities (PP) for BI and 1,000 bootstrap (BP) resampling for ML and MP. Haplotype networks were constructed based on alignment of different genes of 61 *M. tornieri* via the median-joining (MJ) method in POPART v1.7.^79^^,^^87^ Discriminant analysis of principal components (DAPC) was performed using the R package adegenet v2.1.11^84^ based on alignment of 61 *M. tornieri* mitogenomes without *CR2*, testing K-values from 2 to 8. Based on the same alignment, analysis of molecular variance (AMOVA) and pairwise *F*st calculations were performed between samples from different localities and areas in Arlequin v3.5.^80^

Mantel tests were performed using the R package vegan v2.7.1^85^ on 60 georeferenced samples to assess isolation by distance (IBD), where great-circle geographic distances were calculated using the R package geosphere v1.5.20,^86^ and genetic p-distances based on the alignment of mitogenomes without *CR2* were computed using MEGA v11.0.13. Additionally, we performed tests on samples from northern and central Tanzania separately. As three individuals from central Tanzania (central Tanzania divergent clade, CTDC) showed considerable divergence, potentially biasing the results, we repeated the analyses excluding these individuals for both Northern+Central Tanzania and Central Tanzania datasets.

We used DnaSP v6.0.7 to calculate haplotypes, haplotype diversity(*h*), nucleotide diversity (*Pi*), and average nucleotide differences (*K*) for all localities and area samples based on alignment of mitogenomes without *CR2*. To facilitate comparisons, *Pi* was also calculated for all samples and for the northern and central Tanzania samples based on the 13 PCGs. Given that CTDC may cause the overestimation of *Pi* in central Tanzania, we recalculated *Pi* for this area excluding CTDC. For comparison, we computed *Pi* of the 13 PCGs for ten other chelonian species using NCBI data, selected for their larger number of individual sequences. For sequences lacking annotation, PCGs were annotated using the same procedure as described above for *M. tornieri*. To account for potential bias from genetic substructure, we calculated *Pi* separately for each ESU in species with genetic substructure.

#### Maternal demographic history in Tanzania

To infer the maternal demographic history of the *M. tornieri*, we performed neutrality tests (Tajima’s *D* and Fu’s *Fs*) using Arlequin 3.5 and estimated the nucleotide mismatch distribution under a constant population size model using DnaSP v6.0.7, based on alignment of the 61 *M. tornieri* mitogenomes without *CR2*.

We also conducted Bayesian skyline plot (BSP) analysis using the same alignment. After determining the optimal nucleotide substitution model (Table S9) under the AICc criterion using PartitionFinder v2.0, we performed two independent runs in BEAST 1.8.4^81^ under a strict molecular clock, assuming a mutation rate of 1.005 × 10^-9^ per site per year for the turtle mitogenome.^40^ Each run consisted of 20,000,000 generations, with sampling every 1,000 generations. The results were combined using LogCombiner v2.6.4^82^ and convergence was assessed in Tracer v1.6.1^83^ by confirming that all parameters had an effective sample size >200, after discarding the first 25% as burn-in. The final BSP plots were generated in Tracer v1.6.1.

BSP analyses were also repeated separately for individuals from Northern and Central Tanzania to investigate the demographic history of *M. tornieri* in different areas. In addition, to avoid potential bias in Central Tanzania caused by CTDC individuals, we performed an analysis excluding this group.

#### Differentiation analysis between Tanzania and Kenya populations

We further downloaded *COX1* sequences of two Kenyan *M. tornieri* (MK545068, MK545069) and two outgroups, *I. elongata* (DQ080043/NC_007695) and *I. forstenii* (DQ080044/NC_007696) (Table S7), to investigate whether the Kenyan population is genetically distinct from the Tanzanian population. After alignment using MAFFT v7.313, all *COX1* sequences were trimmed based on sequence homology to ensure equal length, and ML and BI phylogenetic analysis were conducted using the same methods previously applied to the mitogenome without *CR2* within the Tanzanian population. Haplotype networks were constructed based on alignment of *COX1* of 63 *M. tornieri* via the MJ method in POPART v1.7.

Given the considerable genetic divergence inferred between Tanzanian and Kenyan populations of *M. tornieri*, we further estimated their divergence time. To improve accuracy and provide time references, mitochondrial sequences from 54 turtle species/subspecies were retrieved from NCBI based on the time framework established in a previous study,^41^ with only one representative individual of *M. tornieri* from each of the Tanzanian and Kenyan populations being selected (Table S8). Using alignment and model selection methods as previously described, trimmed homologous sequence alignments and optimal substitution models were obtained (Table S9). Divergence time was inferred via BI in BEAST v1.8.4, employing a Yule tree prior and a relaxed molecular clock with a lognormal distribution. Four fossil calibration points validated by previous study^41^ were applied, with lognormal prior distributions parameterized by offset, mean, and standard deviation (SD): 1) an offset of 50.3 million years ago (Mya) for the divergence between Testudinidae and Geoemydidae (Mean=25.4, SD=0.5); 2) 33.9 Mya for the crown Testudinidae (Mean=16, SD=0.5); 3) 33.9 Mya for the crown Testudininae (Mean=6, SD=0.6); and 4) 11.8 Mya for the divergence between *Chelonoidis carbonarius* and *C. denticulatus* (Mean=10.75, SD=0.5). Two independent MCMC runs were performed, each for 200 million generations with sampling every 1,000 generations. Convergence was assessed by confirming that all parameters had ESS greater than 200 and the first 20% of trees were discarded as burn-in using Tracer v1.6.1. The two independent runs were combined using LogCombiner v2.6.4, and the final maximum clade credibility tree was generated with TreeAnnotator v2.6.4.^82^

### Quantification and statistical analysis

In this study, samples sizes from 11 localities across northern and central Tanzania (Figure 1) were as follows: Sino (n = 7), Besi (n = 7), Tarangire-Poachers Hide (n = 4), Tarangire-Chemchengeu (n = 2), Tarangire-Matete (n = 1), Vilima Vitatu (n = 11), Tampori (n = 1), Kinyasi (n = 8), Mlua (n = 2), Kolo (n = 7), Jenjeluse (n = 10). Mitogenomes were assembled using GetOrganelle v1.7.5,^66^ NOVOPlasty v4.3.5,^67^ and PMAT v2.1.5,^64^ with structural validation through read mapping using BWA-MEM v0.7.18^68^ and Samtools v1.21.^69^ The assembled mitogenomes were annotated using MITOS v2.1.9^70^ with *tRNA* predictions cross-referenced with tRNAscan-SE v1.21^71^ (Figure 2). *Pi*, *h*, *K*, and Ka/Ks ratios were calculated in DnaSP v6.0.7.^74^ Phylogenetic trees were constructed using maximum likelihood in IQ-TREE v2.2.2^75^(1,000 bootstraps for node support), Bayesian inference in MrBayes v3.2^76^ (posterior probabilities for node support) (Figure 3), with optimal partitioning and substitution models selected by PartitionFinder v2.0.^78^ Phylogenetic tree was also constructed using maximum parsimony method in MEGA v11.0.13^77^ (Figure S3). Haplotype networks were generated using the median-joining method in POPART v1.7.^79^ DAPC was conducted in adegenet v2.1.1^84^ (testing K = 2–8). AMOVA, pairwise *F*st, and neutrality tests (Tajima’s *D* and Fu’s *Fs*) were performed in Arlequin v3.5^80^ (significance tested with 10,000 permutations, *p* < 0.01 for *F*st) (Table 1). IBD was assessed via Mantel tests in vegan v2.7.1^85^ (significance based on 999 permutations). Demographic history was inferred using mismatch distributions in DnaSP v6.0.7^74^ and BSP in BEAST v1.8.4^81^ under a strict molecular clock (Figure 5). Divergence times between Tanzanian and Kenyan populations were estimated in BEAST v1.8.4^81^ using a relaxed clock with lognormal distribution, Yule prior, and four fossil calibrations (Figure 6). The convergence of all MCMC used in the analyses was verified with Tracer v1.6.1,^83^ ensuring ESS of parameters > 200. All statistical analyses incorporated multiple independent runs where applicable, and significance levels were set at *p* < 0.05 (indicated by ∗) unless otherwise specified.

## Supplemental information

Document S1. Figures S1–S5

Table S1. Information on *Malacochersus tornieri* sample

Table S2. Information on the assembled mitogenome of the *Malacochersus tornieri*

Table S3. Start and stop codons of protein-coding genes in the mitogenome of *Malacochersus tornier*

Table S4. Pairwise Fst of *Malacochersus tornieri* populations across locations The pairwise *F*st among locations within Northern Tanzania and those within Central Tanzania are highlighted in green and blue, respectively. “+” indicates statistically significant and “-” indicates not statistically significant. Significance level = 0.01.

Table S5. AMOVA results for *Malacochersus tornieri* samples across locations and areas

Table S6. *Pi* calculated from 13 protein-coding genes across different chelonian species Sequences highlighted in red and blue denote distinct ESUs within species, for which *Pi* was estimated separately.

Table S7. Information on COX1 used for genetic structure analysis of the *Malacochersus tornieri* populations in Kenya and Tanzania

Table S8. Information on COX1 used for divergence time estimation of the *Malacochersus tornieri* populations in Kenya and Tanzania

Table S9. The subset partitions, best models, and sites used in phylogenetic analysis, Bayesian skyline plot, and divergence time estimation The values enclosed in parentheses following protein-coding genes indicate the positions of codons.
