# Supplementary material for: Mitogenomes reveal multiple evolutionary units and low genetic diversity of the critically endangered pancake tortoise Malacochersus tornieri
Source: iScience. 2026 Feb 25;29(3):115142. doi: 10.1016/j.isci.2026.115142 (PMC12996709; doi:10.1016/j.isci.2026.115142)
Supplement: Document S1. Figures S1–S5 [file mmc1.pdf]

iScience, Volume *29*

Supplemental information

Mitogenomes reveal multiple evolutionary units and low genetic diversity of the critically endangered pancake tortoise *Malacochersus tornieri*

Chuan Jiang, Nassoro Mohamed, Rudolf Mremi, Xuda Liu, Gabriel Mayengo, Yang Liu, Reginald T. Mwaya, Wenwen Zhu, Yiming Gao, and Bo Li

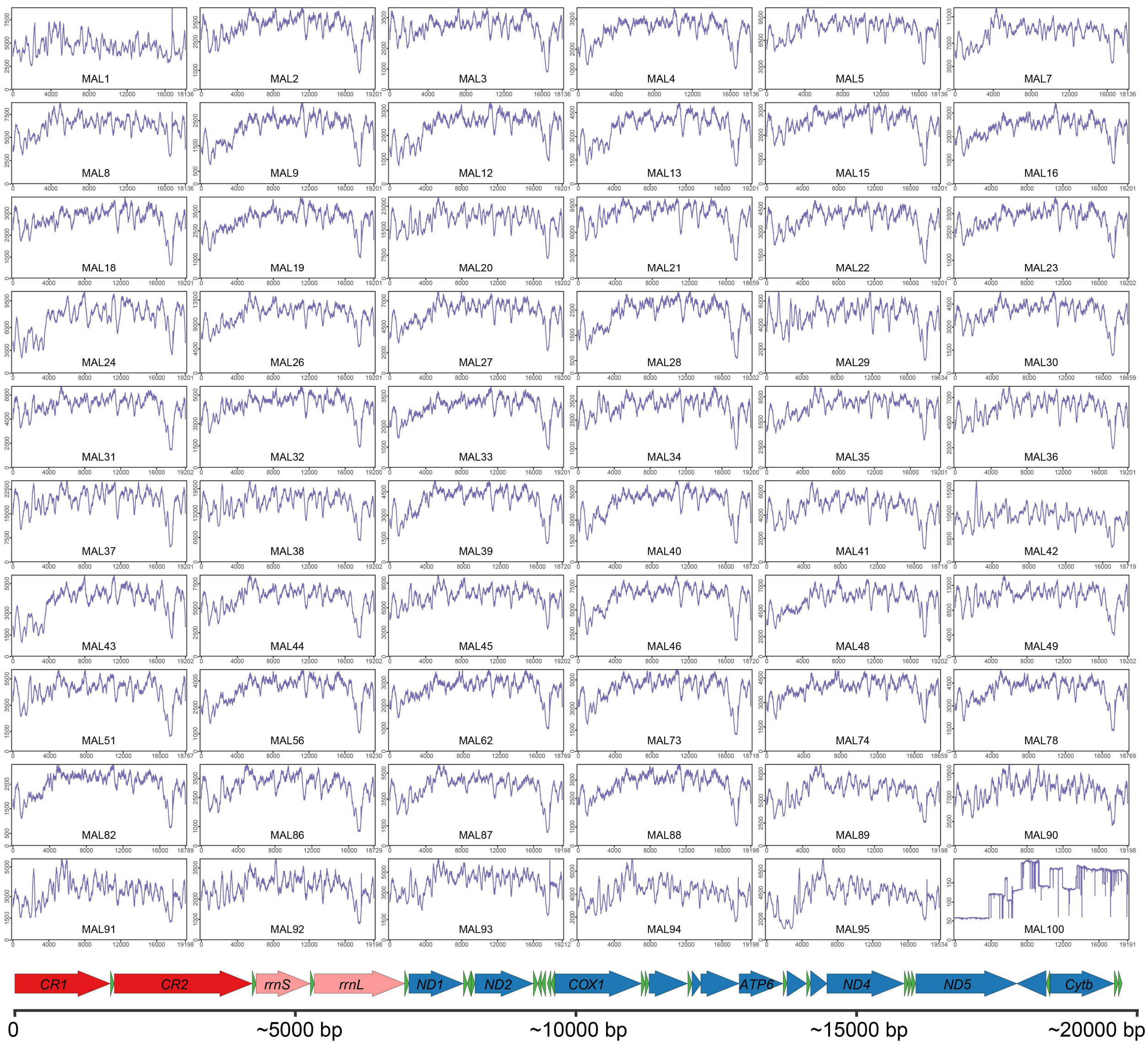

Figure S1. Sequencing depth line plots, generated by mapping reads to their respective mitogenomes assembled from the same data, reflect the completeness of each assembly. Except for MAL100, which was assembled using HiFi long-read data, all other samples were assembled using PE150 short-read data. Below the depth plots, a schematic annotation shows the positions of genes along the mitogenome.

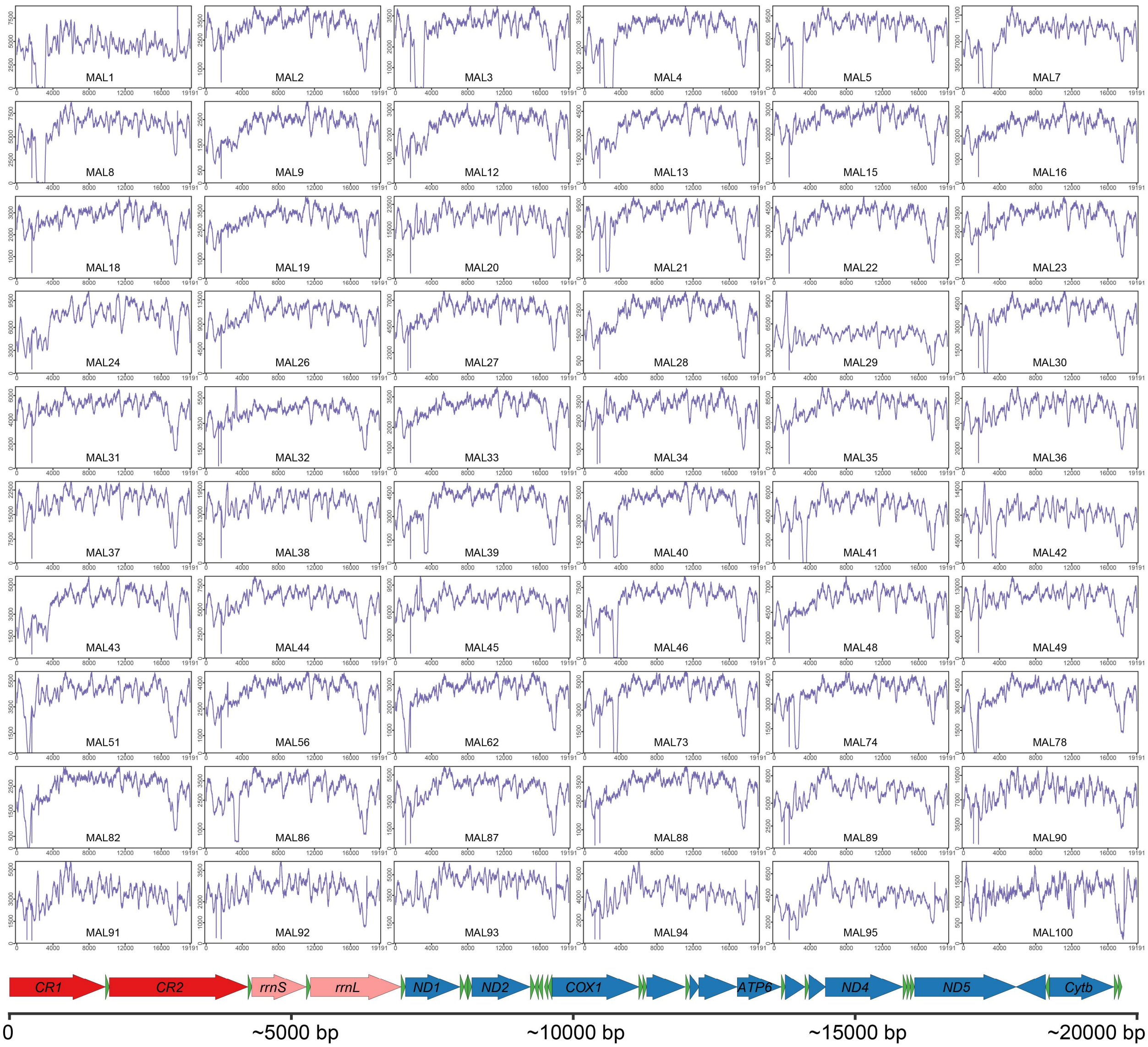

Figure S2. Line plot of sequencing depth from each sample's PE150 short reads mapped to the MAL100 HiFi-assembled mitogenome, revealing variation in the two control regions in some samples. Below the depth plots, a schematic annotation shows the positions of genes along the mitogenome.

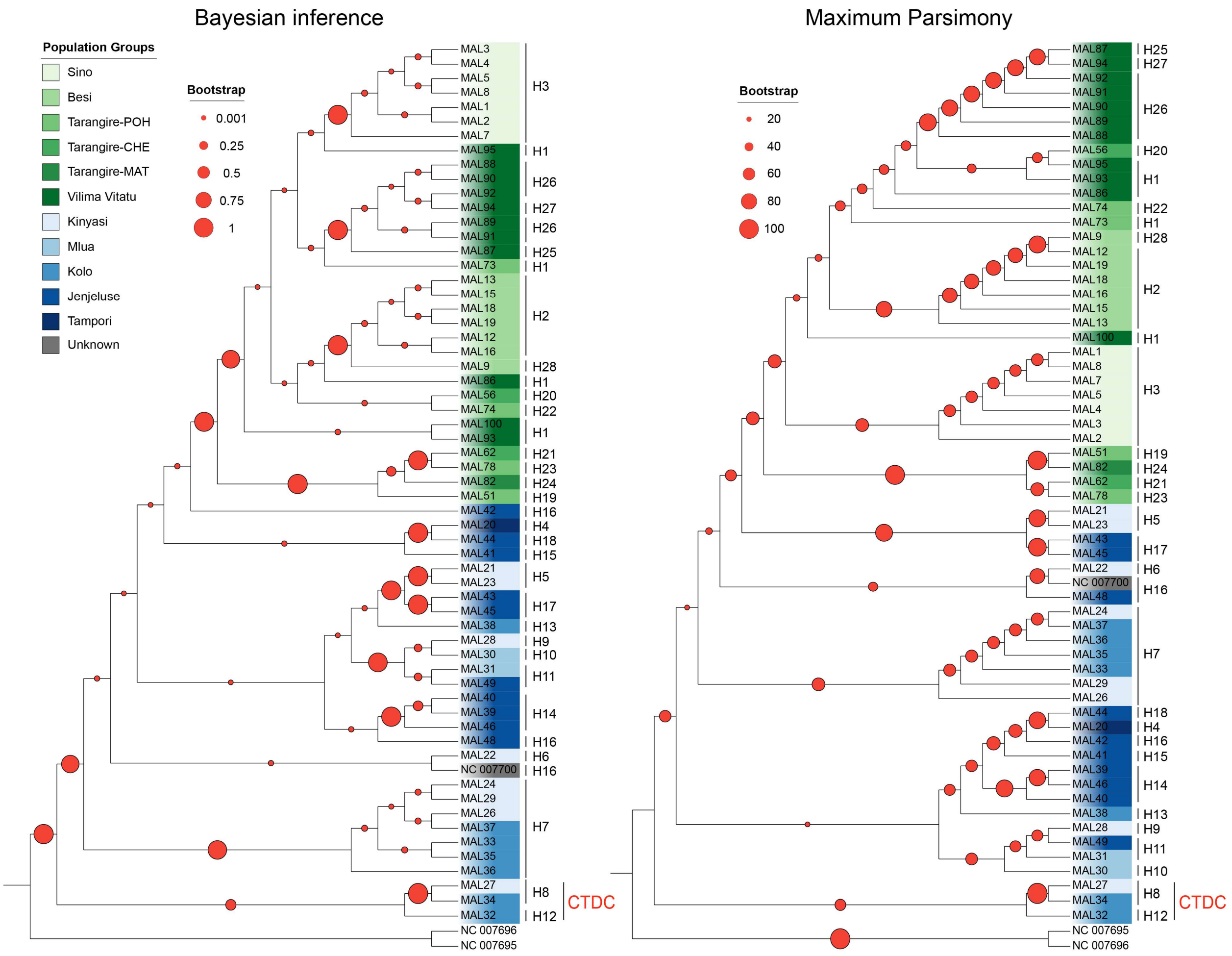

Figure S3. Individual-based phylogenetic tree inferred from mitogenome without the CR2. A.) ML phylogenetic tree. B.) MP phylogenetic tree. Haplotypes are listed on the right side of the sample labels. The red-marked CTDC represents three highly divergent samples.

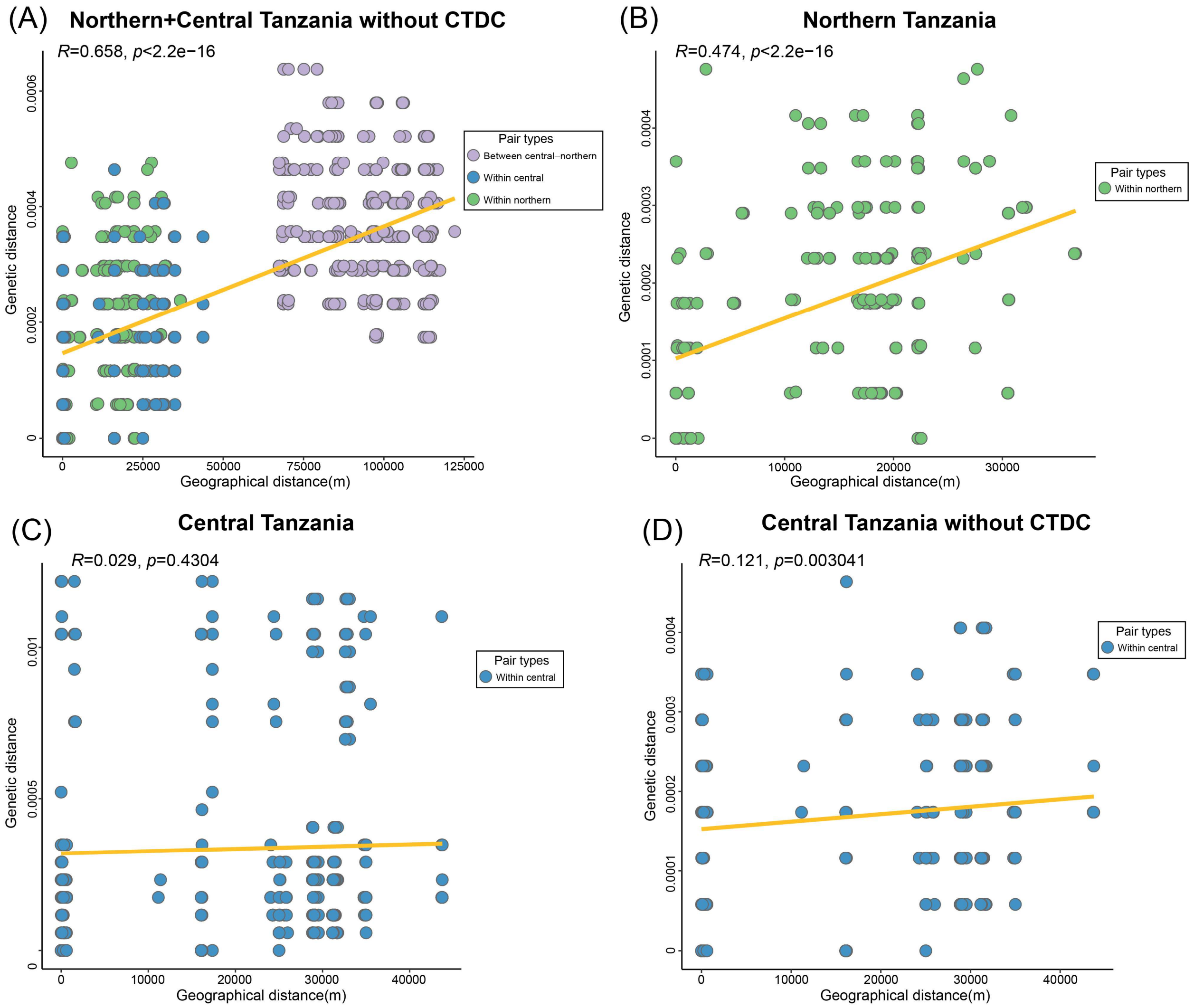

Figure S4. Mantel test for individuals of the *M. tornieri* from different areas. Except for the Mantel test involving all individuals from Central Tanzania (which showed no statistical significance), the other three tests revealed a significant positive correlation between genetic and geographic distances in *M. tornieri*. Green and blue dots denote sample pairs from northern and central Tanzania, respectively, while purple dots represent interregional pairs.

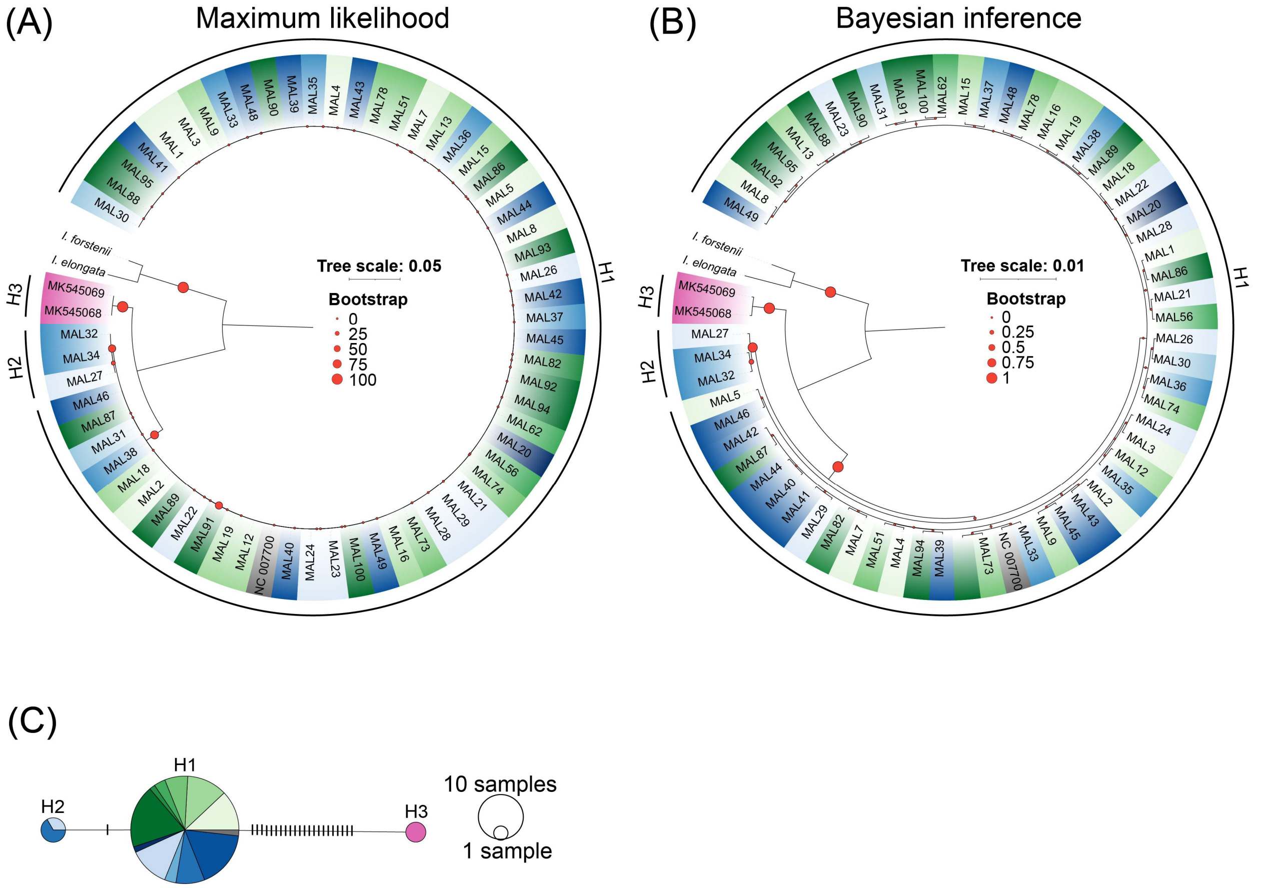

Figure S5. Phylogenetic analysis and haplotype network reveal a deep genetic split between Tanzanian and Kenyan populations of *M. tornieri*. A.) Phylogenetic tree reconstructed using the maximum likelihood method based on partial *COX1* sequences. B.) Phylogenetic tree reconstructed using Bayesian inference based on partial *COX1* sequences. C.) Haplotype network constructed using the median-joining method
